# Assessment of Knowledge of and Attitude Toward Oral Cancer Among the Outpatient Population in a Tertiary Care Rural Hospital

**DOI:** 10.7759/cureus.36637

**Published:** 2023-03-24

**Authors:** PB Anirudh, Susanna TY, Sagayaraj A, Ravishankar S

**Affiliations:** 1 Department of Otorhinolaryngology, Sri Devaraj Urs Academy of Higher Education and Research, Kolar, IND; 2 Department of Biochemistry, Sri Devaraj Urs Academy of Higher Education and Research, Kolar, IND; 3 Department of Community Medicine, Sri Devaraj Urs Academy of Higher Education and Research, Kolar, IND

**Keywords:** oral cancer screening, health education & awareness, rural population, knowledge assessment, oral cavity cancer

## Abstract

Introduction

Lack of knowledge and awareness about oral cancer and its risk factors and negligence of the early warning signs play a crucial role in raising the incidence of the disease. Therefore, the aim of this present study is to assess the awareness of oral cancer among the local population regarding the prevalence, etiology, early signs of oral cancer, and treatment options available for the same.

Materials and methods

The study was approved by the institutional ethics committee. A cross-sectional study was done on 158 patients from 15-70 years. The questionnaire comprised closed-ended questions to assess the subject’s awareness, knowledge, and attitude toward the prevalence, causative factors, early signs that suggest oral cancer, and treatment options available for oral cancer.

Results

The study population consisted of 61% females and 39% males; the participants were aged between 15 and 70 years and the majority of them belonged to the 46-60-year age group (39.2%). Most of the participants (46%) had completed secondary education. Thirty-two point nine percent (32.9%) had not heard about oral cancer, 43.7% considered tobacco chewing and smoking risk factors, but only 25.8% were aware of early signs of oral cancer. Individuals who were unaware of oral cancer were educated.

Conclusion

This is a simple method to understand the participants' knowledge of oral cancer and its risk factors. Based on the results, we can identify the populations that are unaware of oral cancer, and they can be educated regarding early screening, prevention, and control.

## Introduction

Oral cancer is a malignancy arising from the lips or oral cavity. The majority of oral cancers histologically originate from squamous cells so it is known as oral squamous cell carcinoma. They account for nearly 48% of head and neck cancer. The burden of oral cancer in India is huge accounting for 77,000 new cases, with 52,000 deaths reported annually, which is approximately one-fourth of the global incidence. The important causative factors of this disease are alcohol consumption, tobacco abuse, genetic predisposition, old age, immunodeficiency, a diet deficient in fruits and vegetables, and viral infections such as human papillomavirus and herpes virus [1,2].

In rural India, there is an increased prevalence of tobacco usage by both men and women. In light of this, it is anticipated that there would be a significant incidence and prevalence of oral precancerous and cancerous lesions. Cigarette smoke weakens the immunity in the oral cavity by causing gingivitis, periodontitis, and oral cancer. In certain rural areas still, issues like poverty, illiteracy, poor health care, poor knowledge, and lack of awareness about oral health, oral hygiene, and the prevalence of oral cancer or lack of knowledge of oral cancer are prevalent [3].

In our region, the most common site of head and neck cancer among both genders is the oral cavity, with the highest incidence of 29.6% of all cancers reported and treated at our institution. The incidence of oral cancer reported at our institution was 28.7% in males and 30.3% in females; the survival rate of these patients is poor due to their late presentations [4].

Public awareness about oral cancer risk factors, early signs, and lifestyle modifications will increase the possibility of disease identification. Early detection and prevention are the key challenges in the fight against oral cancer because the stage at diagnosis is an important factor, as it indirectly influences the prognosis. Screening and early diagnosis are crucial in identifying the disease in its earliest stages [5].

Therefore, the aim of this present study is to assess the awareness of oral cancer among the local population regarding the prevalence, etiology, early signs of oral cancer, and the treatment options available for the same.

## Materials and methods

This was a descriptive study carried out from May to July 2022. The study was approved by the institutional ethics committee. A total of 158 patients attending the screening OPD were enrolled in the study (IEC NO: DMC/KLR/IEC/201/2021-22).

This was a questionnaire-based assessment of the outpatient population at our tertiary rural care hospital attached to a medical college (Appendices). A structured questionnaire in English consisted of relevant questions. It was pretested, necessary alterations were done, and it was distributed to subjects at the screening OPD. An information sheet regarding the study questionnaire was given to the subjects and informed consent was obtained from every participant before the distribution of the questionnaire. The questionnaire comprised closed-ended questions to assess the subject’s awareness, knowledge, and attitude about the prevalence, causative factors, early signs that suggest oral cancer, and treatment options available for oral cancer. Inclusion criteria were age 15 to 70 years, any gender, patients visiting the outpatient department, and people from rural areas. Exclusion criteria were patients already diagnosed with oral cancer or precancerous lesions, patients not willing to participate in the study, and patients from urban areas. The questionnaire was divided into two sections; Section 1 included the sociodemographic details of the participant such as age, gender, marital status, occupation, address, and education level was recorded. Section 2 consisted of closed-ended questions regarding subject knowledge and attitude toward oral cancer. The results of the survey are expressed in percentages.

## Results

The study population consisted of 61% females and 39% males. The participants were aged between 15 and 70 years and the majority of the participants were in the 46-60-year age group (39.2%). Most of the participants (46%) had completed secondary education. Thirty-two point nine percent (32.9%) of participants were not aware of oral cancer, and the majority of them (65.6%) are unaware that the region has the highest incidence of oral cancer. Nearly 43.8% of people say oral cancer is more prevalent among women as compared to men. More than half of the study participants (51%) are not aware of the risk factors for oral cancer, and 44.3 % of people think alcohol as the major causative factor but the majority of them have the habit of tobacco and pan chewing, and they refused to quit their habits. The duly filled questionnaire was collected and evaluated by the authors. Based on the patient’s answers, individuals were educated regarding oral cancer. The results of the survey are depicted in Table 1 and Table 2.

**Table 1:** Demographic details of the study participants

SOCIODEMOGRAPHIC VARIABLE		PERCENTAGE
AGE IN YEARS	15-30	5.7%
	31-45	24.7%
	46-60	39.2%
	>60	30.4%
SEX	MALE	39%
	FEMALE	61%
EDUCATION LEVEL	ILLITERATE	0
	UPTO PRIMARY EDUCATION	27%
	UPTO SECONDARY EDUCATION	46%
	GRADUATE	27%
	POSTGRADUATE	0
RESIDING AREA	RURAL	100%
	URBAN	0

**Table 2:** Subjects' knowledge of and attitude to oral cancer The important risk factors for oral cancer are alcohol consumption, tobacco abuse, genetic predisposition, old age, immunodeficiency, a diet deficient in fruits and vegetables, and viral infection such as human papillomavirus and herpes virus [1].

1) HAVE YOU HEARD ABOUT MOUTH/ORAL CANCER?	A)YES	67.1%
	B)NO	32.9%
2) DO YOU KNOW OUR REGION HAS THE HIGHEST INCIDENCE OF ORAL CANCER COMPARED TO OTHER CANCERS?	A)YES	34.4%
	B)NO	65.6%
3) ORAL CANCER IS PREVALENT AMONG WHICH AGE GROUP?	A)40-60	27.8%
	B)20-40	34.8%
	C)10-20	14.6%
	D)DON’T KNOW	22.8%
4) ORAL CANCER IS PREVALENT AMONG WHICH GENDER?	A)MALES	15.1%
	B)FEMALES	43.8%
	C)BOTH	41.1%
5) DO YOU KNOW THE RISK FACTORS THAT CAN CAUSE ORAL CANCER?	A)YES	49%
	B)NO	51%
6) IF YES, INDICATE THE CAUSES OF ORAL CANCER.	A)SMOKING	12.7%
	B)ALCOHOL	44.3%
	C)PAN CHEWING	31%
	D)POOR ORAL HEALTH	3.2%
	E)OLD AGE	2.5%
	F)INFECTION	1.9%
	G)REDUCED INTAKE OF FRUITS AND VEGETABLES	1.9%
	H)HEREDITARY	2.5%
7) DO YOU HAVE ANY OF THE HABITS LISTED HERE? LIKE:	A)SMOKING	16.5%
	B)TOBACCO/PAN CHEWING	52.5%
	C)ALCOHOL	19%
	D)NONE	12%
8) IF YES ARE YOU WILLING TO QUIT THOSE HABITS?	A)YES	56.3%
	B)NO	43.7%
9) DO YOU THINK ORAL CANCER CAN OCCUR EVEN IN NON-ADDICTS?	A)YES	57.1%
	B)NO	42.9%
10) WHICH OF THE FOLLOWING SIGNS DO YOU THINK ARE THE SIGNS SUGGESTIVE OF ORAL CANCER?	A)WHITE OR RED PATCH IN THE MOUTH	25.8%
	B)RAISED WHITE AND RED PATCHES IN THE MOUTH	22.2%
	C)ULCERS IN THE MOUTH	20.3%
	D)SWELLING IN THE MOUTH	10.8%
	E)PAIN AND BLEEDING FROM THE MOUTH	2.5%
	F)DON’T KNOW	18.4%
11) DO YOU THINK THE DETECTION OF ORAL CANCER IN ITS EARLY STAGES COULD INCREASE THE SUCCESS OF THE TREATMENT?	A)YES	53.8%
	B)NO	46.2%
12) IS ORAL CANCER A CURABLE DISEASE?	A)YES	51.9%
	B)NO	48.1%
13) DO YOU KNOW ABOUT ANY DIAGNOSTIC/SCREENING TESTS (TO ASSESS THE PRESENCE OF CANCER ) WHICH IS DONE FOR ORAL CANCER?	A)YES	43.7%
	B)NO	56.3%
14) IF YES, WHAT ARE THE DIAGNOSTIC TESTS WHICH ARE DONE FOR ORAL CANCER?	A)CLINICAL EXAMINATION	34.2%
	B)DENTAL X-RAY	35.4%
	C)BIOPSY	20.9%
	D)CT	8.2%
	E)MRI	1.3%
15) DO YOU KNOW ANY TREATMENT OPTIONS FOR ORAL CANCER?	A)YES	57.6%
	B)NO	42.4%
16) IF YES, WHAT ARE THE TREATMENT OPTIONS AVAILABLE FOR ORAL CANCER?	A)SURGERY	29.7%
	B)RADIOTHERAPY	25.3%
	C)CHEMOTHERAPY	29.7%
	D)IMMUNOTHERAPY	7.7%
	E)TARGETED THERAPY	1.9%
	F)COMBINATION THERAPY	1.9%
	G)OTHERS, PLEASE INDICATE	3.8%
17) IS ORAL CANCER A CONTAGIOUS DISEASE?	A)YES	62%
	B)NO	38%

## Discussion

Oral cancer is the most common cancer in the head and neck region. Due to poor knowledge of the early symptoms and signs, patients neglect seeking medical treatment and present in an advanced stage where the tumor is inoperable or requires extensive resection or chemotherapy, leading to severe morbidity and mortality. Many surveys and health education are being conducted to promote awareness of oral cancer in the general public, at the institutional level in dental and medical colleges but there are very few surveys taking place in rural areas, which should be given due importance [6,7].

The study done by Shodan et al. (India) and Razavi et al. (Iran) reported that half of the study population was not aware of oral cancer [8,9]. In the present study, 67.1% of participants said they have heard about oral cancer but they are unaware of its incidence in the region. This can be attributed to the early age of addiction to alcohol and tobacco, which leads to oral cancer at a later age. In our study, participants have said that at 20-40 years, the incidence of cancer is high and it is more prevalent among women as compared to men but when compared to the activities done among the local population where the maximum cases were in the age group of 60-69 years, cancer of the oral cavity predominated in both genders [4]. This helps us understand that oral cavity cancer has become prevalent among middle-aged men and women due to changes in their lifestyle, increased use of tobacco, alcohol consumption at a young age, and food habits. One study explains the burden of oral cancer in Indian patients belonging to lower socioeconomic groups who lack the attitude to come forward for early medical treatment and have misapprehensions [10]. In the present study, the majority of people were aware of oral cancer, think women are most vulnerable to developing oral cancer than men, and attribute alcohol as the primary causative factor followed by pan chewing and smoking. The majority of the study participants were women (61%) and had the habit of chewing tobacco.

Reddy et al. also reported a lack of awareness regarding the early signs and symptoms of oral cancer among patients attending a dental hospital in Hyderabad [6]. In the present study, 51% of the participants were not aware of the risk factors of oral cavity cancer and they started smoking tobacco and consuming packed tobacco prepared by using tobacco leaves, areca nut, and slaked lime; and around 44.3 % of participants knew alcohol as the major risk factor for oral cavity cancer but, unfortunately, in this region, the oral cancer load is due to tobacco chewing [4]. In the present study, 52.5% of participants had the habit of tobacco chewing and it is more common among women. Forty-three point seven percent (43.7%) were not willing to quit tobacco chewing because they were not aware that tobacco is a causative factor for oral cancer. Nearly 40% of study participants were told about advanced signs of oral cavity cancer and 18.4% did not know the early signs of oral cavity cancer; hence, the rural population was given health education and awareness about tobacco being a major risk factor for oral cancer. Other risk factors like poor oral hygiene, a diet deficient in fruits and vegetables, alcohol consumption, spicy food, and early signs of oral cavity cancer like a white or red patch in the mouth, raised patches, ulcers in the mouth, and swelling in the mouth, was also explained. In the present study, 25.8% of participants thought early signs of oral cancer are white or red patches in the mouth, very few thought they were pain and bleeding from the mouth, and nearly one-fifth of the participants (18.4%) did not know the signs suggestive of oral cancer. When questioned about diagnostic and screening tests to detect the presence of cancer, 53.8% of survey participants believed that early diagnosis of cancer would improve the success of the treatment and said that oral cancer is curable. On the other side of the coin, 46.2% of survey participants were unaware of the early screening and diagnostic tests, hence, this could be a reason for a patient to present to a doctor at an advanced stage of oral cancer. Participants who were aware of the diagnostic and screening tests available to detect the presence of oral cancer thought that a dental X-ray followed by a clinical examination and biopsy is useful in early diagnosis, and few also said that a computed tomography scan and magnetic resonance imaging scan are also useful. The majority of study participants were aware of the treatment options available for oral cancer - 29.7% said surgery followed by radiotherapy and chemotherapy, and 3.8% were not aware of the treatment options available.

The study's first limitation is the limited number of participants, as only patients who were attending the hospital's outpatient department were able to take part. Second, because physicians created the questionnaire, it was prepared from their perspective rather than the participants. The questionnaire was written in English, but participants requested that the questions be translated into their regional language. The degree of whatever bias this may have induced into the study is yet unknown. The outcomes might alter depending on the language and response. Finally, the fact that study participants were only allowed a single option for each question may have overestimated their awareness of oral cancer. Future research can be organized with a qualitative study design to solve these constraints even if the study's findings reflect the existing reality.

Health education programs must be imparted in a more structured manner like teaching programs right from the school level. Mass media must be used to educate the public about the risk factors and the harm caused by tobacco use, betel quid chewing, and alcohol use, along with the premalignant changes of oral cancer. Also, our people should be motivated to go for regular oral examinations so that the premalignant changes can be identified and diagnosed as early as possible. Health education must be divided according to different groups like school students, the general public, and cancer patients. Using this method in each group, specific problems can be addressed, and we can provide better health support, education, and awareness. Frequent health camps should be conducted, questionnaires should be distributed and the responses should be evaluated. Patients or the public with the least score should be identified and educated on the same day either through a group discussion or one-to-one communication. This helps in creating awareness in the camp. Anganwadi teachers, village health nurses, health inspectors, primary health care physicians, and dental surgeons should initiate and organize low-cost educational programs so the underprivileged group in our society is benefitted. The prime duty of the treating physician is not only restricted to providing the best treatment but also to educating the patient and attendant appropriately.

## Conclusions

According to the findings of this study, oral cancer and precancer are not widely known in society, and as a result, people only seek medical care after the cancer is already advanced. The majority of participants are unaware of the risks, symptoms, or current treatments for oral precancer and cancer. Early detection of oral cancer carries a good prognosis. People who live in distant locations must be encouraged to take part in camps, and the entire community must get health education about the risk factors for oral cancer and how to stop young people from consuming tobacco in all forms. This is necessary to bring change in their knowledge and attitude. The assessment made regarding the knowledge and attitude of oral cancer among the local population in this study will help in implementing an effective public awareness program on the risk factors of oral cancer and thereby reducing the incidence rate of oral cancer in the future.

## Figures and Tables

**Figure 1:**
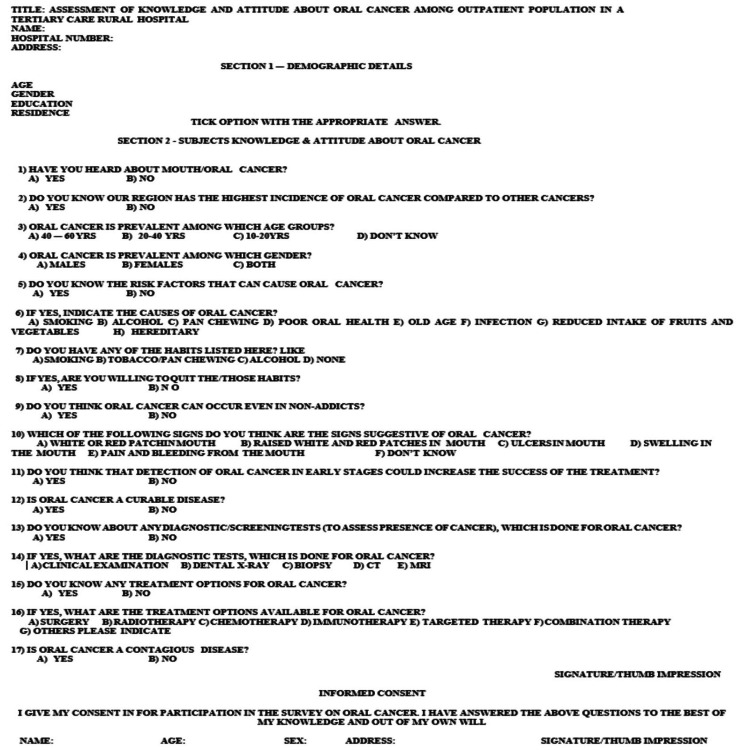
Questionnaire designed for the participants, including the patients' consent
